# Moral wiggle room and group favoritism among political partisans

**DOI:** 10.1093/pnasnexus/pgae307

**Published:** 2024-10-15

**Authors:** Andrea Robbett, Henry Walsh, Peter Hans Matthews

**Affiliations:** Department of Economics, Middlebury College, Middlebury, VT 05753, USA; Department of Economics, Middlebury College, Middlebury, VT 05753, USA; Department of Economics, Middlebury College, Middlebury, VT 05753, USA; Department of Economics, Aalto University School of Business and Helsinki Graduate School of Economics, Helsinki 02150, Finland

**Keywords:** identity, group bias, moral wiggle room, social preferences, affective polarization

## Abstract

How does the availability of excuses for self-interested behavior impact group favoritism? We report the results of a preregistered experiment, conducted on the eve of the 2022 midterm elections, in which American political partisans made payoff distribution choices for themselves and a partner who was known to be a co-partisan or opposing partisan. Under full information, participants exhibit significant group favoritism. However, when the payoff consequences for one's partner are initially hidden, participants exploit this excuse to act selfishly regardless of who their partner is and ignorance rates are identical for in-group and out-group members. As a result, moral wiggle room has a significantly larger impact on selfish behavior for those interacting with co-partisans than opposing partisans, leading to a reduction in group favoritism.

**Significance Statement**

We report the results of an experiment designed to assess how group favoritism interacts with the availability of excuses for self-interested behavior among American political partisans. We find that partisans are more likely to exploit excuses to act selfishly when matched with co-partisans than with opposing partisans. This suggests that group favoritism among partisans is not just due to caring more about outcomes experienced by members of one's in-group, but that normative or image-based considerations play a major role. We also document differences in the use of moral wiggle room across partisan groups.

## Introduction

How does the availability of excuses for self-interested behavior impact group favoritism? Everyday experience and several decades of social science research indicate that people regularly act more altruistically toward members of their own social groups (1, 2). This *group favoritism* can manifest even when group identities are artificially constructed in the lab on the basis of essentially meaningless characteristics, such as whether people underestimate the number of dots appearing on a computer screen or prefer paintings by Klee over Kandinsky (1, 3), but is strongest when rooted in naturally occurring identities (4, 5). Political affiliation, in particular, has emerged as one of the most salient social identities for many Americans (6), and the last four decades in American politics have been characterized by increasing dislike and distrust between rank and file Democrats and Republicans. This *affective polarization* is largely distinct from ideological or issue-based polarization and manifests in everyday interactions outside the political domain (7, 8).

There is now considerable evidence that people treat opposing partisans worse than co-partisans in a wide range of settings. Several recent studies have found that partisans give more to members of their own party in dictator and trust games and contribute more in public goods games with co-partisans (4, 5, 7, 9). Kranton et al. (4) further find that individuals vary in their intrinsic “groupiness” and that “groupy” individuals are both more likely to identify with a political party and to exhibit in-group favoritism even in minimal groups established on the basis of aesthetic preferences. Other studies have found that partisans’ in-group favoritism extends outside the context of economic games. Partisans set lower reservation wages when the employer shares their affiliation (10), prefer to award scholarships to co-partisans (7), are less likely to interview opposing partisans for jobs in a resume audit study (11), are less willing to choose opposing partisans for work tasks (12), and are more open to matching with co-partisans on a dating platform (13). This differential treatment can manifest as favoritism toward co-partisans, antipathy for opposing partisans, or both.

The tendency to treat people differently based on who they are or how they have previously acted is typically modeled by assuming that people care about the material outcomes experienced by others, and the magnitude of this concern shifts depending on with whom they interact (2, 14). Yet a rapidly growing literature in experimental economics over the past two decades demonstrates that the availability of excuses for self-interested behavior can also dramatically influence people's choices, suggesting that image concerns and the desire to adhere to social norms play a role alongside outcome-based preferences (15–19). In particular, when the mapping from choices to outcomes is ambiguous, individuals may be tempted to exploit this “moral wiggle room” to both make selfish choices and preserve their self- or social-image as someone who is fair. For example, participants who must choose an allocation of payments between themselves and a partner are less likely to be generous when they can remain willfully ignorant of how their choice affects their partner, which is the paradigm used in our experiment (15, 16). Furthermore, choices become less generous when dictators have plausible deniability because the recipient is not sure whether the decision was randomly implemented by the computer instead (15, 18); dictators can opt out of the dictator game entirely and take the pie for themselves (17, 20); or dictators can take money in addition to giving, such that giving nothing is not the most selfish option on the menu (19, 21). While neutrally framed laboratory games with clear incentives typically admit little room for excuses, such ambiguity and the opportunities for moral reasoning are prevalent in interactions outside the lab and, thus, are essential for understanding real-world interactions between partisans.

In this paper, we ask how moral wiggle room interacts with political identities and group favoritism. We report the results of a preregistered online experiment, which uses the hidden information paradigm initially developed by Dana et al. (15) to study the exploitation of moral wiggle room. In our experiment, American political partisans chose how to allocate payments to themselves and a partner. The decision-maker either did not have information about their partner's political affiliation or knew their partner to be a co-partisan or opposing partisan. To increase the salience of partisan identity, the experiment was conducted in the hours leading up to the closing of the polls in the 2022 midterm elections, since there is evidence that individuals identify more strongly with their party as an election approaches (22). In a baseline condition with full payoff information, participants chose between an allocation that gives both participants the same moderate payment and an allocation that provides the decision-maker with somewhat higher earnings while giving their partner very little. Consistent with the existing literature, participants in this baseline condition were significantly more likely to act selfishly when matched with an opposing partisan than a co-partisan (with choices among those who did not know the affiliation of their partner falling midway in between).

In a separate, hidden information, condition, participants knew their own payment from the two options, but were initially unaware which option was associated with the higher payment for their partner. However, they could readily and costlessly reveal this information with the click of a button. Consistent with Dana et al. (15) and subsequent replications (16, 23), when affiliations were unknown, most participants elected to remain ignorant and the proportion of selfish choices significantly increased relative to the full information condition. We find that partisans also exploit moral wiggle room when they know they are matched with an opposing partisan or co-partisan—however, they do so at different rates. While people make more selfish choices overall when matched with an opposing partisan, the increase in selfishness when wiggle room is introduced is very similar to those who did not know affiliations, both around 21 percentage points. However, when matched with a co-partisan, the effect of wiggle room on selfishness is even greater (around 32 percentage points). With respect to ignorance rates, about two-thirds of participants avoided learning the consequences of their choice for their partner and this did not vary with their partner's identity. However, the one-third of decision-makers who did inform themselves were more likely to choose selfishly when matched with an out-group member. The end result is that in-group bias *decreases* with the introduction of moral wiggle room: the gap in selfish choices when matched with an in-group versus out-group member is about 21 percentage points with full information, but approximately halved (to under 10 percentage points) when an excuse for selfish behavior is available.

## Experimental design

We recruited 1698 American political partisans (798 Republicans and 900 Democrats) on Prolific in the days leading up to the 2022 United States midterm elections (Monday, November 7, and Tuesday, November 8).^a^ After providing their political affiliation and answering questions about the strength of their partisan identity, participants were informed that they would be randomly matched with another participant and make a binary choice that would determine the monetary payoff they and their partner received. In all cases, the participant chose between option A, which provided them with $1.80 or option B, which provided them with a payoff of $1.50. However, the information they had about their partner's identity and payoffs varied by treatment, as described below.

We used a 3 × 3 experimental design, in which we independently varied both the partisan identity of their partner and what they knew about their partner's payoffs. The design and number of participants in each treatment cell, as well as the breakdown by Democrats and Republicans, are shown in Tables S1 and S2. First, participants were randomly assigned to one of the three partner-type conditions: they either knew their partner shared their political affiliation (Co-partisan), knew their partner was affiliated with the opposing party (Opposing Partisan), or were not given any information about their partner's political affiliation (Unknown).

Second, we randomly varied the information that participants received about the payoffs of their partner. Following Dana et al. (15), participants were randomly assigned to one of the three payoff treatments. In the baseline Full Information—Conflicting Payoffs condition, participants knew that option A maximized their own payoff by giving them $1.80, but gave their partner only $0.30, while option B gave both people $1.50.^b^ Therefore, the two players’ interests conflict: option A maximizes the decision-maker's payoff, while option B maximizes their partner's payoff as well as their collective payoff and eliminates any inequality in their earnings. Screenshots of this choice, and the choices under the other two information treatments, are included in Fig. S1.

In the other two information treatments, the other player's payoff was initially hidden. The participant knew that one option paid their partner $0.30, while the other option paid $1.50, and that each option was equally likely to be the one associated with the higher amount for their partner. That is, they knew that there was a 50% chance that the payoffs were the same as those in the full information game, in which case their incentives conflicted, and a 50% chance that their incentives were aligned, and option A left both players better off. While this information was initially hidden, the participant could costlessly reveal their partner's payoff with a single click prior to making their choice. Specifically, the participant could either choose option A or option B without learning the impact on their partner, or they could select “Reveal” and see the full payoff table while making their choice.

Participants who were not in the full information treatment were randomly assigned to either the Hidden Information—Conflicting Payoffs treatment or the Hidden Information Aligned Payoffs treatment. Note that the experience of the decision-maker would be identical in these two treatments if they chose not to reveal the information.

On average, participants spent just over 5 min in the experiment (median = 4.2 min), including instructions. They were paid a base rate of $1 and received an average bonus of $1.39, which translates to an average hourly wage over $28. Participants knew that they were playing the game as the decision-maker and were also serving in the role of recipient in a parallel game with a different partner and that a coin toss would determine which game counted for their payoff. The base payment was received immediately upon completion while the bonuses were promised (and paid) within 48 h of completion of the study.

As discussed in the preanalysis plan, there are two approaches to analyze the impact of moral wiggle room in a hidden information design. Dana et al. (15) compare the proportion of selfish choices with full information to those when information is hidden but the underlying payoffs conflict (regardless of whether the participant knows this). This approach drops the data from the Hidden Information—Aligned Payoff condition, which serves only to ensure that there is no deception in the experimental instructions that inform subjects that both types of payoffs for their partner are equally likely. In contrast, Grossman and Van der Weele (16) compare the proportion of selfish choices in the full information condition with the ignorance rate across both hidden information conditions. In what follows, we analyze the data using both approaches, in line with our preregistration plan, which can be found at: https://aspredicted.org/54W_MZ3.

## Results

**Fig. 1.**
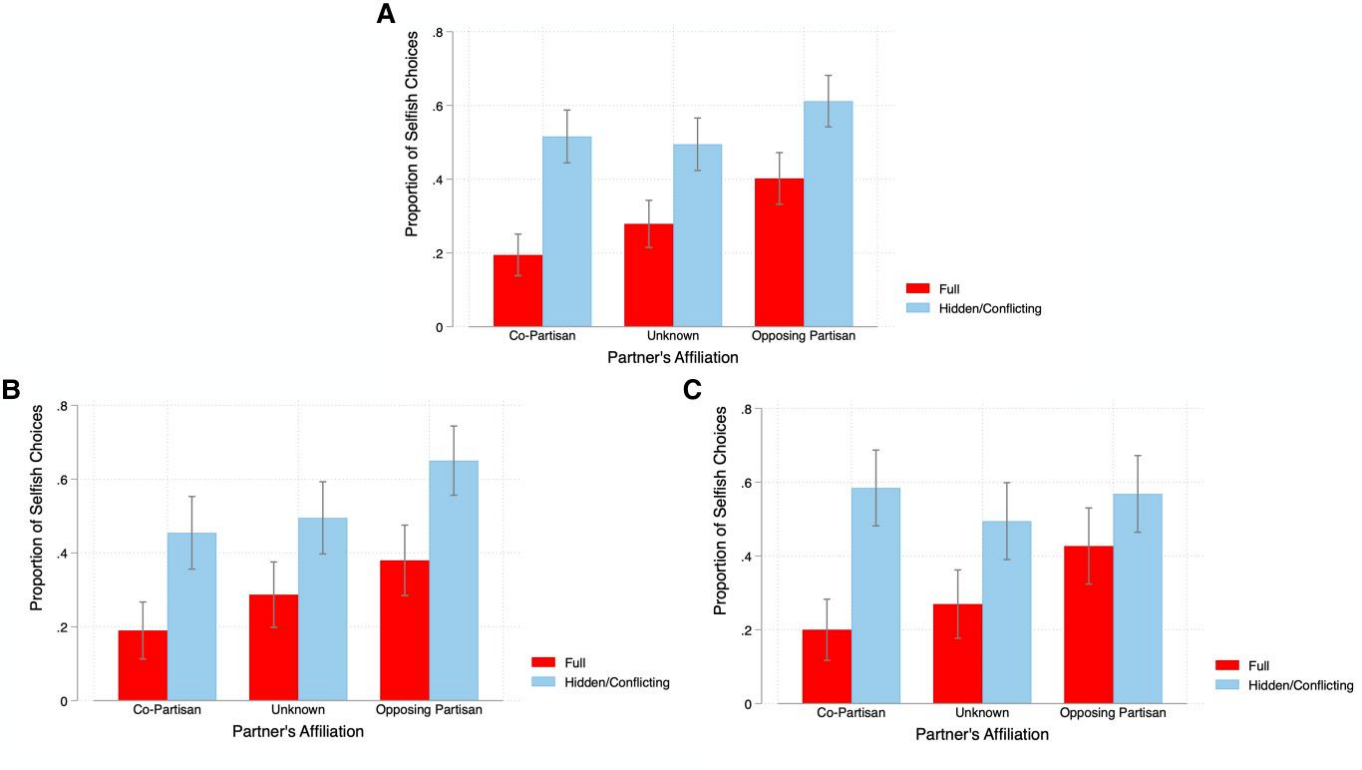
The proportion of participants choosing the “selfish” option A by partner type in the Full Information—Conflicting Payoffs and Hidden Information—Conflicting Payoffs treatments. A) All participants; B) only Democrats; and C) only Republicans.

To begin, we consider whether choices in the baseline Full Information—Conflicting Payoffs treatment vary by partisan affiliation of the recipient.^c^ The dark red bars in panel A of Fig. 1 show the proportion of participants choosing the self-interested option A when there was no possibility of remaining ignorant of the consequences. In this treatment, there is a clear evidence of group favoritism: while <20% of participants made the self-interested choice when their partner shared their affiliation, twice as many (40.2%) acted selfishly when matched with an opposing partisan (*P* < 0.001, *Z* = 4.407). Furthermore, choices fall in between these two extremes when their partner's affiliation was not known (27.89%), which suggests that both in-group favoritism and out-group animus are present.^d^

Having replicated the prior results on partisan favoritism within our experimental environment, we turn to our main question of whether this in-group favoritism influences partisans’ willingness to exploit moral wiggle room. Following the approach of Dana et al. (15), we first compare the proportion of selfish choices in the Full Information treatment (the dark red bars in panel A of Fig. 1) with those in the Hidden Information—Conflicting Payoffs treatment (light blue bars, same panel), when the participants face the same underlying payoffs but may not necessarily learn this information. When partisan affiliation is unknown, our results are qualitatively similar to the existing literature: the proportion of selfish choices increases from 27.89 to 49.47% (*P* < 0.001, *Z* = 4.313), indicating that 21.58% of our participants exploit moral wiggle room (as encoded by the gap in the dark red and light blue bars in panel A of Fig. 1). The similarity of the Unknown treatment with the previous work (that did not provide information about the recipient's identity) provides a check for our results and, going forward, we focus on the Co-Partisan and Opposing Partisan treatments.^e^

We likewise observe participants exploiting moral wiggle room when they have information about their partner, both when they know that they are matched with an opposing partisan or with a co-partisan. While people make more selfish choices overall when matched with an opposing partisan, the *increase* in selfishness when wiggle room is introduced is very similar (20.96 *pp*, *P* < 0.001, *Z* = 4.06) to those who did not know affiliations (21.58 *pp*). However, when matched with a co-partisan, the effect of wiggle room on selfishness is even greater (32.12 *pp*, *P* < 0.001, *Z* = 6.52). The end result is that the group bias *decreases* with the introduction of moral wiggle room: the gap in selfish choices when matched with an opposing partisan versus a co-partisan is 20.7 *pp* with full information but is approximately halved (9.58 *pp*, *Z* = 1.869, *P* = 0.06) when partisans can avoid payoff information.^f^

We formalize these results in the regression framework reported in Table 1, which considers the data from the treatments in which the payoffs were not aligned and the participants knew the partisan affiliation of their partner. Model 1 regresses an indicator for whether the participant made the selfish choice on indicators for being in the Hidden Information—Conflicting Payoffs treatment (with Full Information as the omitted condition) and for being matched with a co-partisan (with Opposing Partisan as the omitted condition), along with their interaction and demographic controls. The negative coefficient on Co-Partisan confirms that, in the absence of wiggle room (and controlling for demographics), partisans are over 20 percentage points less likely to choose selfishly when they are matched with a member of their own party. The positive coefficient on Hidden Information confirms that, with controls in place, wiggle room increases selfishness when matched with an opposing partisan by nearly 20 percentage points. Finally, the positive interaction between the Co-Partisan and Hidden Information conditions indicate that *more* people exploit moral wiggle room when matched with a co-partisan.

**Table 1.** Selfish choice when payoffs conflict.

	(1)	(2)	(3)	(4)	(5)
	All	Democrats	Republicans	Strong	Weak
Co-partisan	−0.221^c^	−0.199^c^	−0.257^c^	−0.366^c^	−0.120^b^
	(0.0454)	(0.0631)	(0.0670)	(0.0717)	(0.0593)
Hidden info	0.196^c^	0.266^c^	0.116	0.153^a^	0.211^c^
	(0.0506)	(0.0695)	(0.0756)	(0.0782)	(0.0677)
Co-partisan × hidden info	0.129^a^	0.00672	0.266^c^	0.181^a^	0.107
	(0.0687)	(0.0961)	(0.100)	(0.105)	(0.0915)
Strong partisan	0.0557	0.0422	0.0652		
	(0.0355)	(0.0475)	(0.0548)		
Female	0.00661	0.0320	−0.0160	0.0412	−0.0330
	(0.0346)	(0.0476)	(0.0527)	(0.0523)	(0.0465)
Democrat	−0.0524			−0.0778	−0.0503
	(0.0365)			(0.0568)	(0.0482)
Age	−0.00269^b^	−0.00135	−0.00370^a^	−0.00325^a^	−0.00204
	(0.00133)	(0.00184)	(0.00196)	(0.00195)	(0.00185)
College graduate	0.0831^b^	0.0360	0.134^b^	0.0625	0.101^b^
	(0.0355)	(0.0488)	(0.0525)	(0.0552)	(0.0471)
White	0.0470	0.0421	0.0714	−0.00937	0.0716
	(0.0423)	(0.0542)	(0.0736)	(0.0655)	(0.0561)
Rural	−0.102^a^	−0.0695	−0.127^a^	−0.0841	−0.120^a^
	(0.0545)	(0.0820)	(0.0749)	(0.0875)	(0.0704)
City	−0.0568	0.00384	−0.126	−0.0656	−0.0401
	(0.0507)	(0.0644)	(0.0859)	(0.0779)	(0.0671)
Suburb	−0.0405	0.0300	−0.113^a^	0.00218	−0.0767
	(0.0434)	(0.0608)	(0.0630)	(0.0630)	(0.0597)
Constant	0.475^c^	0.340^c^	0.543^c^	0.677^c^	0.398^c^
	(0.0805)	(0.107)	(0.114)	(0.132)	(0.108)
Observations	755	399	356	327	428

Robust standard errors in parentheses.

^a^ *P* < 0.10. ^b^*P* < 0.05. ^c^*P* < 0.01.

We next consider whether there are heterogeneous effects by political affiliation. Panels B and C of Fig. 1 break down the results in panel A by political party, and reveal that the interaction effect between wiggle room and group membership is driven entirely by the Republicans in our sample. While panel B of Fig. 1 indicates that Democrats exploit wiggle room at a similar rate regardless of their partner's affiliation, panel C shows wiggle room has very little impact on Republicans’ behavior when matched with an opposing partisan, but increases their likelihood of making the selfish choice by nearly 40 *pp* when matched with a co-partisan. These heterogeneous effects are confirmed in Models (2) and (3) in Table 1, which report the same specification as Model (1) for Democrats and Republicans, respectively. Both Democrats and Republicans exhibit in-group favoritism under full information (as indicated by the negative coefficient on Co-Partisan). Democrats increase their selfishness by 26.6 *pp* (*P* < 0.001) in the presence of wiggle room when matched with an opposing partisan, and this increases insignificantly when instead matched with a co-partisan (0.0067, *P* = 0.944). In contrast, Republicans do not take much advantage of wiggle room when matched with a Democrat (0.116, *P* = 0.126), but sharply increase their selfishness by an additional 26.66 *pp* (*P* = 0.008) when the person for whom they can avoid learning the payoff consequences is a co-partisan rather than opposing partisan. Models (4) and (5) consider only those who strongly identified as a Democrat or Republican, respectively, and indicate that these patterns are even more stark among strong partisans. In Table S4, we break down columns (4) and (5) of Table 1 by party affiliation.

We note, too, that whatever demographic characteristics matter for the participant's choice in the overall sample—in particular, age, college degree, and rural location—reflect their substantial influence on Republican but not Democratic respondents. Consider, for example, the relationship between education and selfish choices. In the combined sample, being a college graduate is predicted to increase the likelihood of selfish behavior 8.31 *pp* (*P* = 0.019), a substantial difference, but the effect for Republicans is much larger, 13.4 *pp* (*P* = 0.007), while that for Democrats is both smaller and insignificant (3.6 *pp*, *P* = 0.458). In a similar vein, there is some evidence that both older and rural Republicans are less selfish, while this pattern does not hold for their Democratic counterparts.

We next turn to the approach taken by Grossman and Van der Weele (16), which includes the data from the Hidden Information—Aligned Payoffs condition and compares the proportion of selfish choices in the Full Information—Conflicting payoffs condition with the rate at which participants remain ignorant in both of the hidden information conditions.

**Fig. 2.**
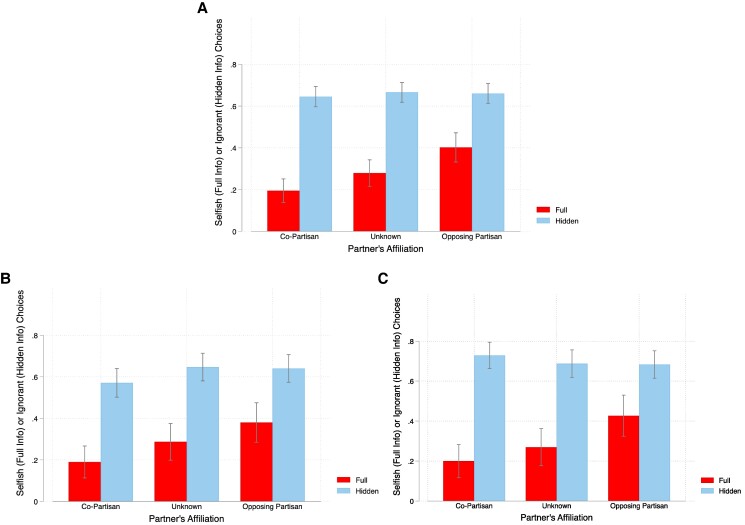
The proportion of participants choosing the “selfish” option A in the Full Information—Conflicting Payoffs treatment or remaining ignorant in either of the Hidden Information treatments. A) All participants; B) only Democrats; and C) only Republicans.

These outcome variables are graphed side-by-side for each partner type in panel A of Fig. 2. Furthermore, this graph reveals one reason why wiggle room has a greater impact when matched with a co-partisan: ignorance rates are essentially identical across partner types (*P* > 0.55 across the three pairwise comparisons). Combined with the baseline in-group bias exhibited in the presence of full information, this implies that the gap between the selfishness rate with complete information and the ignorance rate with hidden information is largest when matched with a co-partisan (49.4 *pp*, *P* < 0.001, *Z* = 10.11) and smallest when matched with an opposing partisan (25.8 *pp*, *P* < 0.001, *Z* = 5.86). The results are formalized in the regressions presented in Table 2 and broken down by political party in panels B and C of Fig. 2. Both confirm that this alternative measure of the exploitation of moral wiggle room coincides with our previous conclusions: (i) there is a significant interaction between moral wiggle room and in-group membership; (ii) this interaction effect is primarily driven by Republicans in our sample; and (iii) these patterns are most pronounced among strong partisans. In Table S5, we break down columns (4) and (5) of Table 2 by party affiliation.

**Table 2.** Selfish choice or ignorance.

	(1)	(2)	(3)	(4)	(5)
	All	Democrats	Republicans	Strong	Weak
Co-partisan	−0.214^c^	−0.189^c^	−0.243^c^	−0.360^c^	−0.107^a^
	(0.0454)	(0.0631)	(0.0670)	(0.0705)	(0.0592)
Hidden info	0.252^c^	0.265^c^	0.253^c^	0.147^b^	0.329^c^
	(0.0434)	(0.0603)	(0.0636)	(0.0680)	(0.0557)
Co-partisan × hidden info	0.201^c^	0.122	0.275^c^	0.299^c^	0.127^a^
	(0.0572)	(0.0804)	(0.0824)	(0.0882)	(0.0751)
Strong partisan	0.000540	−0.0153	0.0168		
	(0.0289)	(0.0396)	(0.0426)		
Female	−0.0194	0.0286	−0.0683^a^	−0.0176	−0.0260
	(0.0280)	(0.0392)	(0.0406)	(0.0430)	(0.0374)
Democrat	−0.0851^c^			−0.0924^b^	−0.0973^b^
	(0.0294)			(0.0459)	(0.0386)
Age	−0.00123	0.00117	−0.00336^b^	−0.000226	−0.00215
	(0.00110)	(0.00156)	(0.00154)	(0.00157)	(0.00153)
College graduate	0.0445	0.0470	0.0339	0.0144	0.0655^a^
	(0.0284)	(0.0403)	(0.0400)	(0.0441)	(0.0375)
White	0.0233	−0.00458	0.0774	0.0338	0.000000886
	(0.0353)	(0.0460)	(0.0567)	(0.0572)	(0.0452)
Rural	−0.143^c^	−0.0682	−0.193^c^	−0.148^b^	−0.139^b^
	(0.0447)	(0.0675)	(0.0592)	(0.0704)	(0.0577)
City	−0.0545	−0.0268	−0.0439	−0.0475	−0.0602
	(0.0409)	(0.0541)	(0.0643)	(0.0643)	(0.0533)
Suburb	−0.0654^a^	−0.0204	−0.104^b^	−0.0699	−0.0595
	(0.0342)	(0.0505)	(0.0468)	(0.0516)	(0.0461)
Constant	0.520^c^	0.325^c^	0.596^c^	0.604^c^	0.487^c^
	(0.0688)	(0.0917)	(0.0977)	(0.110)	(0.0931)
Observations	1,131	598	533	498	633

Standard errors in parentheses.

^a^ *P* < 0.10. ^a^*P* < 0.05. ^b^*P* < 0.01.

**Fig. 3.**
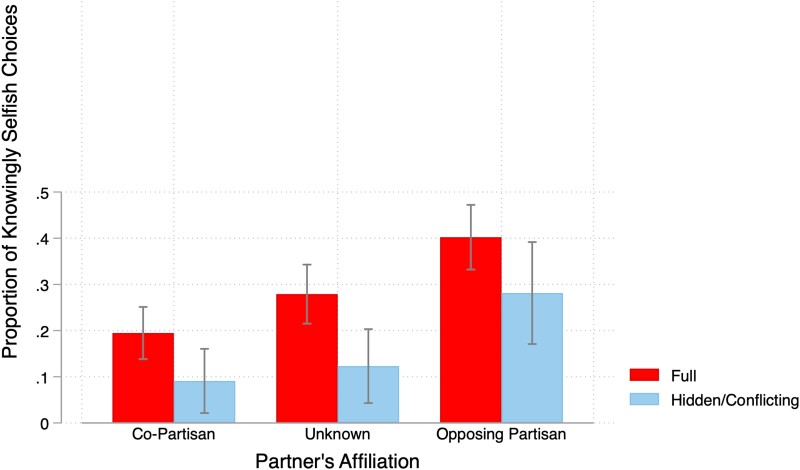
The proportion of participants knowingly choosing the “selfish” option A by partner type in the Full Information—Conflicting Payoffs and after revealing information in Hidden Information—Conflicting Payoffs treatments.

Finally, Fig. 3 considers how likely participants are to choose selfishly when they know that the incentives are conflicting. In this case, we see a clear selection effect: people are significantly less likely to choose selfishly once they have voluntarily learned the consequences for the other person. With controls in place, participants matched with a co-partisan are about 9.67 *pp* (*P* = 0.036) less likely to choose selfishly after they have chosen to reveal information rather than simply being presented with the consequences. This selection effect is similar for opposing partisans (13.13 *pp*, *P* = 0.05). In addition, even those who voluntarily select into receiving information about their partner's payoff consequences are still significantly more likely to go on to choose selfishly when matched with an opposing partisan rather than a co-partisan (20.2 *pp*, *P* = 0.004 with controls). Furthermore, the in-group bias exhibited among those who actively selected information about their partner's payoff is very similar (19 *pp*, *P* < 0.005, *Z* = 2.78) to those with full information (20.7 *pp*, *P* < 0.001). Hence, even though ignorance rates are very similar regardless of partner type (as we saw in Fig. 2), those who do learn the information still go on to favor co-partisans in their distribution choices, which is why the magnitude of in-group favoritism is roughly halved in the hidden information condition but does not disappear entirely.^g^

## Discussion

The theoretical literature on moral decisions and image concerns, of which the exploitation, in equilibrium, of moral wiggle room is a canonical example, provides a natural framework for understanding our results. In the unifying framework of Bénabou et al. (27), the benefits that an individual derives from moral action—in this context, choosing the generous or prosocial allocation—are both direct and image based. The direct benefits depend on the magnitudes of the external benefits and private costs, both of which are fixed in our protocol, as well as two individual- and perhaps context-specific measures. The first, which is not central to our application, is self-control or willpower, which drives a wedge between ex ante costs, and costs at the moment of decision. The second, which is central, is the intrinsic valuation of external benefits, and it easiest to think of two types of individuals, the selfish, for whom external benefits do not matter, and the prosocial, who have (some) intrinsic motivation.

Image benefits in Bénabou et al. (27) are assumed to be proportional to the individual's type, conditional on the choice of action, where, in this setting, the factor of proportionality could be individual and context specific. Without external observers in our setting, these benefits are associated with self-, and not social-, image concerns: it is as though the individual has two selves, separated in time, one of whom looks back, with imperfect recall, after choosing some action. For example, in a pooling equilibrium in which both selfish and prosocial individuals choose the lopsided allocation, the former will nevertheless derive some self-image benefits.

This suggests that in the full information conditions, the likelihood of moral action increases with both its intrinsic value and the importance of self-image, which enhances the understanding of the differential treatment of co-partisans and opposing partisans. One now familiar explanation is to treat this as a difference in intrinsic motivation: our participants cared more about co-partisans than unknown partners, and still more than opposing partisans. But a second, complementary, explanation now emerges: that participants cared more about their self-image when matched with co-partisans than opposing partisans, and this provides a natural point of departure for future research on affective polarization and self-image.

This in turn prompts some reflection on the exercise of moral wiggle room in our data. Bénabou et al. (27) see moral wiggle room as one manifestation of the avoidance of moral choice, in which it is information that is avoided. Their model predicts that individuals will “avoid the ask” when image concerns are high enough, or willpower is low enough. Comparison of the full and hidden information conditions then suggests a prominent role for image concerns, and is consistent with the claim that such concerns were match-specific and mattered more in the case of co-partisans than opposing partisans. Such concerns also facilitated the emergence of a disquieting sort of “equal treatment,” in which participants were selfish with all sorts of partners when provided with the excuse. This leads to further research questions about the formation and evolution of self-image in a polarized world.

We note, however, that this argument rests on the interpretation of the (more or less constant across matches) ignorance rates in Fig. 2 as the sum of “pure selfishness” (since those who would choose the selfish option under full information have little reason to acquire information when it is hidden) and the exercise of image-driven moral wiggle room. From this perspective, it is the match-specific *difference* between ignorance rates under hidden information and rates of selfishness under full information—that is, the difference between the dark red and light blue bars in Fig. 2—that rationalizes our observation.

The recent work by Exley and Kessler (28) argues that other explanations of the “avoidance-selfishness gap” are possible, however, and finds that image-related concerns are important but smaller than once supposed. If, as one reviewer observed, one instead interprets constant ignorance rates as evidence that image concerns are not match-specific, one must either conclude that partisan decisions are not salient at this point—which in turn requires that individuals are somewhat myopic—or that there are equal but offsetting pressures at work.

Last, it remains to be seen whether our results extend beyond partisan politics. Is it the case, for example, that membership in other in-groups comes with expectations that at least some would prefer to evade?

## Conclusion

We report the results of an experiment designed to assess the extent to which moral wiggle room influences in-group favoritism, using a particularly salient social identity: political affiliation of American partisans on the eve of the midterm elections. We begin by replicating previous work showing that, in the absence of moral wiggle room, partisans exhibit an in-group bias and, in the absence of identity considerations, moral wiggle room influences selfishness.

Having confirmed that our results align with the existing body of work, we turn to the question of how moral wiggle room interacts with this in-group bias. We find that partisans exploit moral wiggle room, regardless of their partner's identity—however, they do so at different rates. While those matched with opposing partisans exploit moral wiggle room at a similar rate to those who do not know their partner's identity, partisans are significantly more likely to exploit moral wiggle room when interacting with a co-partisan.

When given the opportunity, most participants remained willfully ignorant of the consequences of their actions, and this information avoidance did not vary by partner type. The minority who did not remain ignorant behaved less selfishly overall but exhibited a similar degree of in-group favoritism. The end result was that the in-group bias exhibited by political partisans *decreased* with the introduction of moral wiggle room. Finally, we find that the interaction between group bias and moral wiggle room is primarily driven by the Republicans in our sample and the results are most pronounced among strong partisans.

## Supplementary Material

pgae307_Supplementary_Data

## Data Availability

Data and Stata script for producing the graphs and regression tables can be found at the OSF repository (DOI 10.17605/OSF.IO/U3X8W): https://osf.io/u3 × 8w/.

## Notes

a The protocol was reviewed by the Middlebury College IRB and determined to be exempt.

b The original Dana et al. (15) study took place in the lab and the decision maker chose between an option giving them $6, while their partner earned $1 and an option that paid both players $5. We scaled the payoffs down 30% to align more closely with standard incentives for a very brief online study.

c Data sharing plan: If accepted for publication, the de-identified dataset and all code used in the analysis will be posted in a publicly-available repository.

d Those who do not know the identity of their partner are more likely to choose selfishly than those matched with a co-partisan (*P* = 0.054) and less selfishly than those matched with an opposing partisan (*P* = 0.012).

e As a further check on the consistency of our results, we can compare this increase in selfishness in the Unknown condition from 27.89% (with full information) to 49.47% (when information is hidden but underlying payoffs to conflict) with previous work using the same paradigm. Dana et al. (15) find that rates increase from 5*/*19 = 26% to 10*/*16 = 62.5% and using a laboratory sample of *n* = 213, Ahumada et al. (23) find that it increases from 17.4 to 55.1%.

f This finding also broadly relates to the literature on moral wiggle room and reciprocity. Notably, there is mixed evidence that moral wiggle room may have a smaller impact on the behavior of second-movers in a multistage game, who have some evidence regarding how kindly the other player has treated them, suggesting that learning about the other player’s intentions constrains their ability to exploit excuses (24,25). In contrast, Malmendier et al. (26) finds that allowing participants to sort out of a dictator game similarly reduces sharing regardless of whether the recipient previously treated the dictator kindly or unkindly, which is more consistent with our results that participants are no less willing (and sometimes more willing) to exploit moral wiggle room when they have additional information about their partner’s identity.

g There is little evidence of spiteful or competitive behavior among those who reveal payoffs and learn that their incentives are aligned with their partner. In this case, participants who are matched with a co-partisan make the choice that makes both better off 100% of the time while those matched with an opposing partisan do so 96.89% of the time (*P* = 0.147). The proportion of selfish choices by information and partner type is summarized in Table S3.
