# Supplementary material for: Moral wiggle room and group favoritism among political partisans
Source: PNAS Nexus. 2024 Oct 15;3(10):pgae307. doi: 10.1093/pnasnexus/pgae307 (PMC11475467; doi:10.1093/pnasnexus/pgae307)
Supplement: pgae307_Supplementary_Data [file pgae307_supplementary_data.docx]

Moral Wiggle Room and Group Favoritism Among

Political Partisans: Appendix

Andrea Robbett Henry Walsh Peter Hans Matthews

Supplementary Figures and Tables

Payoff Information Type

|  | Full Information | Hidden Information | Hidden Information |  |
| --- | --- | --- | --- | --- |
| Partner Type | Conflicting Payoffs | Conflicting Payoffs | Aligned Payoffs | Total |
| Unknown | 190 | 190 | 187 | 567 |
| Co-partisan | 190 | 188 | 187 | 565 |
| Opposing Partisan | 189 | 188 | 189 | 566 |
| Total | 569 | 566 | 563 | 1,698 |

Table S1: Participants by Condition.

Payoff Information Type

| Partner Type | Full | Hidden - Conflicting | Hidden - Aligned | Total |
| --- | --- | --- | --- | --- |
| Unknown | 101 D; 89 R | 101 D; 89 R | 100 D; 87 R | 302 D; 265 R |
| Co-partisan | 100 D; 90 R | 99 D; 89 R | 99 D; 88 R | 298 D; 267 R |
| Opposing partisan | 100 D; 89 R | 100 D; 88 R | 100 D; 89 R | 300 D; 266 R |
| Total | 301 D; 268 R | 300 D; 266 R | 299 D; 264 R | 900 D; 798 R |

Table S2: Participants by Condition Broken Down by Democrats (D) and Republicans (R).

| Information | Unknown | Co-Partisan | Opposing Partisan |
| --- | --- | --- | --- |
| Full | 0.2789 | 0.1947 | 0.4021 |
| Ignorance | 0.7291 | 0.7563 | 0.8193 |
| Revealed Conflicting | 0.1231 | 0.0909 | 0.2813 |
| Revealed Aligned | 0.9836 | 1.0000 | 0.9688 |

Table S3: Proportion of Self-Interested Choices.

Table S4: Selfish Choice When Payoffs Conflict

|  | (1) | (2) | (3) | (4) |
| --- | --- | --- | --- | --- |
|  | Strong Rep. | Weak Rep. | Strong Dem. | Weak Dem. |
| Co-Partisan | -0.509^∗∗∗^ | -0.170^∗∗^ | -0.304^∗∗∗^ | -0.0560 |
|  | (0.124) | (0.0808) | (0.0886) | (0.0906) |
| Hidden Info | 0.0239 | 0.118 | 0.214^∗∗^ | 0.320^∗∗∗^ |
|  | (0.144) | (0.0904) | (0.0939) | (0.107) |
| Co-Partisan x Hidden Info | 0.407^∗∗^ | 0.235^∗^ | 0.0344 | -0.0380 |
|  | (0.176) | (0.123) | (0.133) | (0.140) |
| Female | -0.0910 | 0.0206 | 0.128^∗^ | -0.0902 |
|  | (0.0949) | (0.0655) | (0.0661) | (0.0721) |
| Age | -0.00315 | -0.00337 | -0.00256 | -0.00000621 |
|  | (0.00338) | (0.00246) | (0.00245) | (0.00271) |
| College Graduate | 0.255^∗∗∗^ | 0.0951 | -0.0240 | 0.112 |
|  | (0.0965) | (0.0645) | (0.0682) | (0.0701) |
| White | -0.0855 | 0.119 | 0.00212 | 0.0303 |
|  | (0.120) | (0.0915) | (0.0794) | (0.0762) |
| Rural | -0.207 | -0.0979 | -0.00517 | -0.198^∗^ |
|  | (0.139) | (0.0897) | (0.119) | (0.114) |
| City | -0.282 | -0.0796 | -0.0126 | 0.0112 |
|  | (0.185) | (0.102) | (0.0858) | (0.0953) |
| Suburb | -0.139 | -0.111 | 0.0343 | -0.00974 |
|  | (0.105) | (0.0799) | (0.0811) | (0.0926) |
| Constant | 0.867^∗∗∗^ | 0.444^∗∗∗^ | 0.512^∗∗∗^ | 0.246 |
|  | (0.190) | (0.147) | (0.152) | (0.158) |
| Observations | 115 | 241 | 212 | 187 |

Robust standard errors in parentheses; ^∗^ *p <* 0*.*10, ^∗∗^ *p <* 0*.*05, ^∗∗∗^ *p <* 0*.*01

Table S5: Selfish Choice and Ignorance Rates

|  | (1) | (2) | (3) | (4) |
| --- | --- | --- | --- | --- |
|  | Strong Rep. | Weak Rep. | Strong Dem. | Weak Dem. |
| Co-Partisan | -0.477^∗∗∗^ | -0.163^∗∗^ | -0.292^∗∗∗^ | -0.0387 |
|  | (0.126) | (0.0803) | (0.0854) | (0.0910) |
| Hidden Info | 0.114 | 0.288^∗∗∗^ | 0.165^∗∗^ | 0.408^∗∗∗^ |
|  | (0.130) | (0.0731) | (0.0816) | (0.0879) |
| Co-Partisan x Hidden Info | 0.432^∗∗∗^ | 0.237^∗∗^ | 0.212^∗^ | -0.0107 |
|  | (0.152) | (0.0993) | (0.111) | (0.116) |
| Female | -0.149^∗∗^ | -0.0372 | 0.0506 | -0.00833 |
|  | (0.0735) | (0.0496) | (0.0543) | (0.0584) |
| Age | -0.00221 | -0.00336^∗^ | 0.00136 | 0.0000723 |
|  | (0.00248) | (0.00197) | (0.00199) | (0.00249) |
| College Graduate | 0.0132 | 0.0436 | 0.0252 | 0.0811 |
|  | (0.0734) | (0.0487) | (0.0561) | (0.0589) |
| White | 0.0526 | 0.0821 | 0.0226 | -0.0594 |
|  | (0.109) | (0.0672) | (0.0681) | (0.0641) |
| Rural | -0.243^∗∗^ | -0.163^∗∗^ | -0.0424 | -0.0850 |
|  | (0.111) | (0.0710) | (0.0940) | (0.0965) |
| City | -0.228^∗^ | 0.0224 | 0.0368 | -0.104 |
|  | (0.131) | (0.0734) | (0.0748) | (0.0789) |
| Suburb | -0.209^∗∗∗^ | -0.0532 | 0.0108 | -0.0577 |
|  | (0.0749) | (0.0595) | (0.0699) | (0.0757) |
| Constant | 0.871^∗∗∗^ | 0.491^∗∗∗^ | 0.338^∗∗∗^ | 0.303^∗∗^ |
|  | (0.172) | (0.124) | (0.128) | (0.139) |
| Observations | 173 | 360 | 325 | 273 |

Standard errors in parentheses; ^∗^ *p <* 0*.*10, ^∗∗^ *p <* 0*.*05, ^∗∗∗^ *p <* 0*.*01

Figure S1: Screenshots depicting the payoffs and interface in the different information conditions. A: The payoffs in the Conflicting Payoffs treatments, as shown to all participants in the Full Information - Conflicting Payoffs treatment and those who revealed information in the Hidden Information - Conflicting Payoffs treatments. B: The decision interface for participants in the Hidden Information conditions prior to revealing information. C: The payoffs in the Hidden Information - Aligned Payoff treatment, as shown to participants in this condition who revealed information. D, E, and F: The same screenshots but for the partisan condition in which the participant is matched with a Democrat.


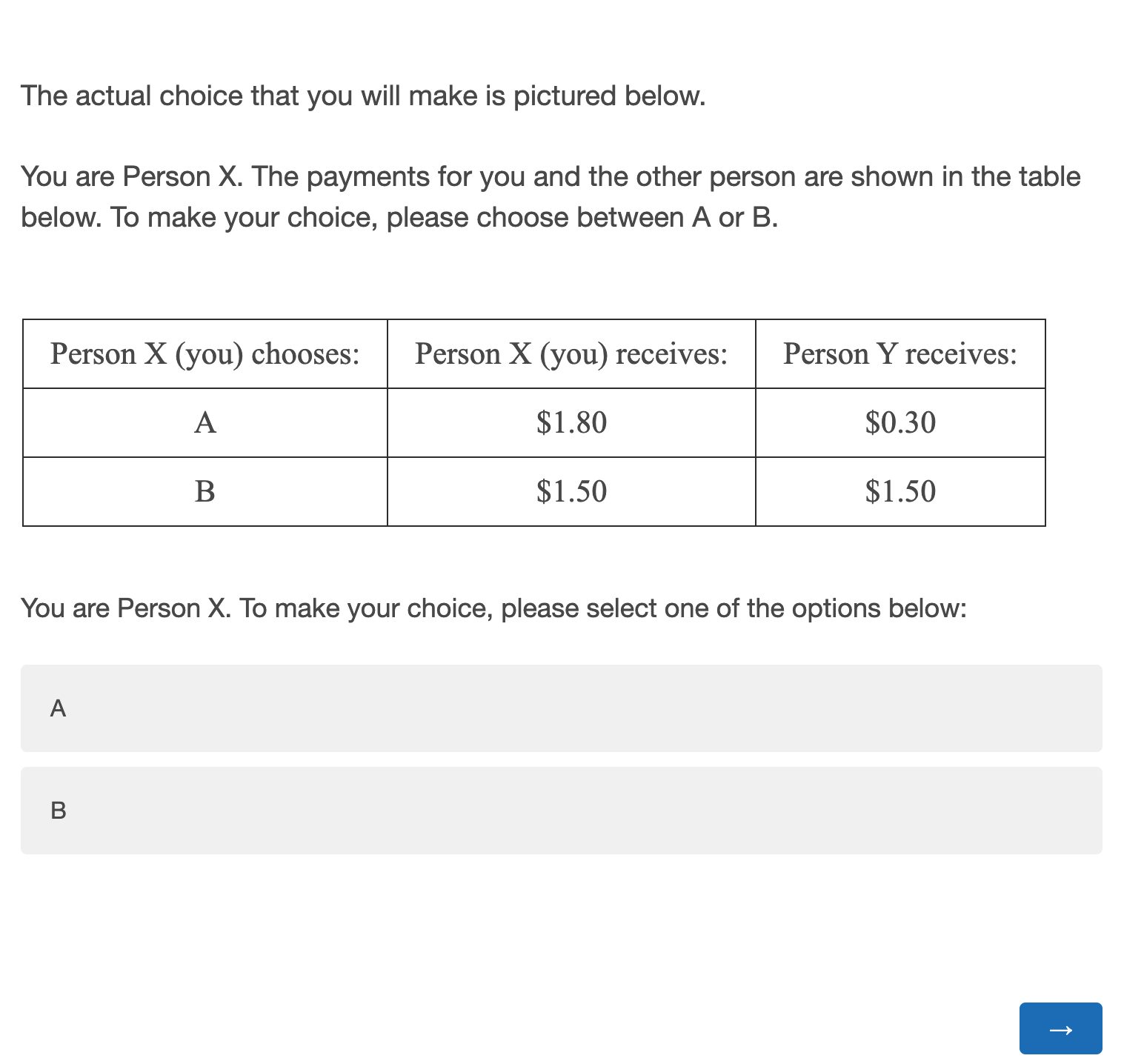


A
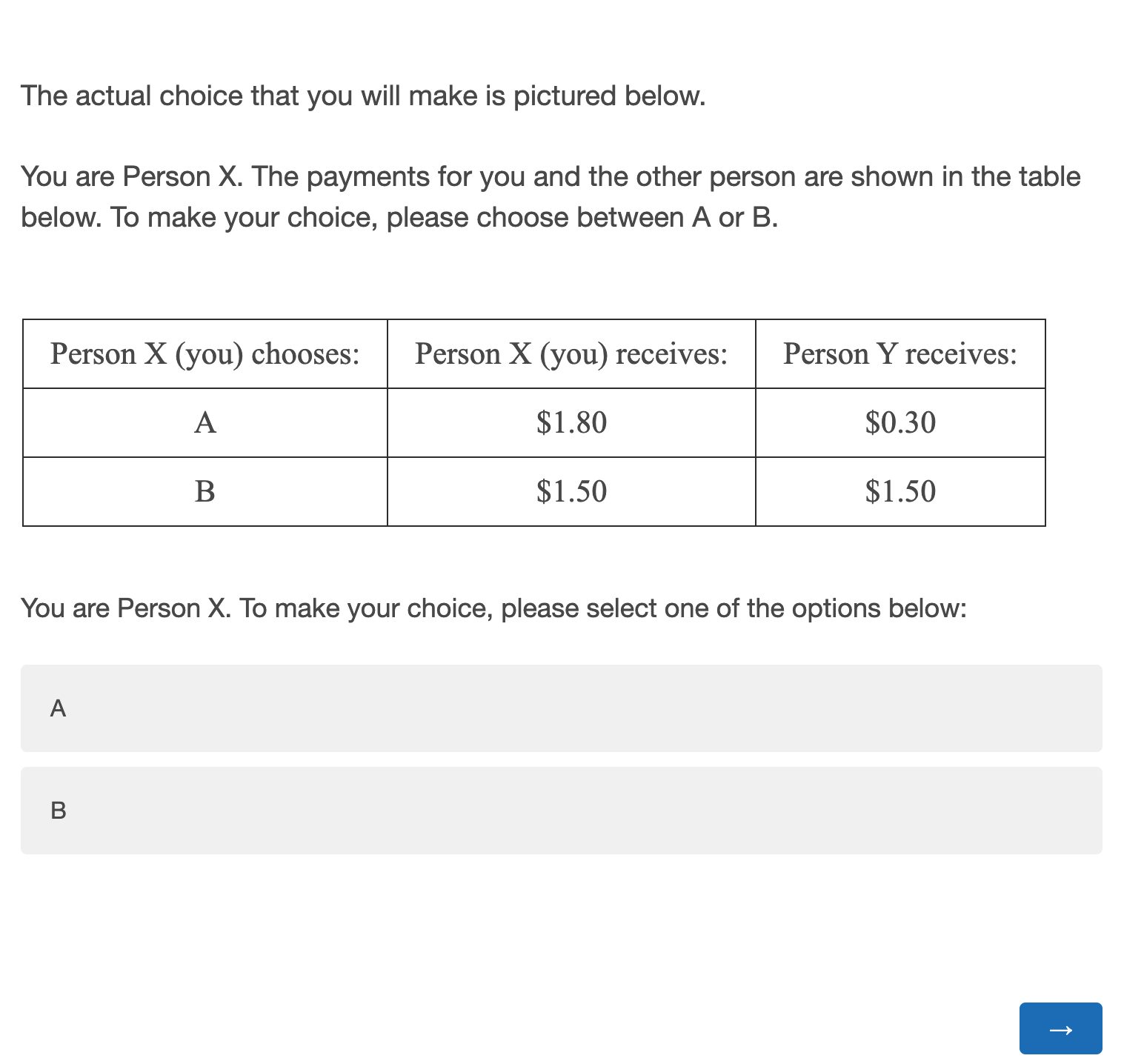


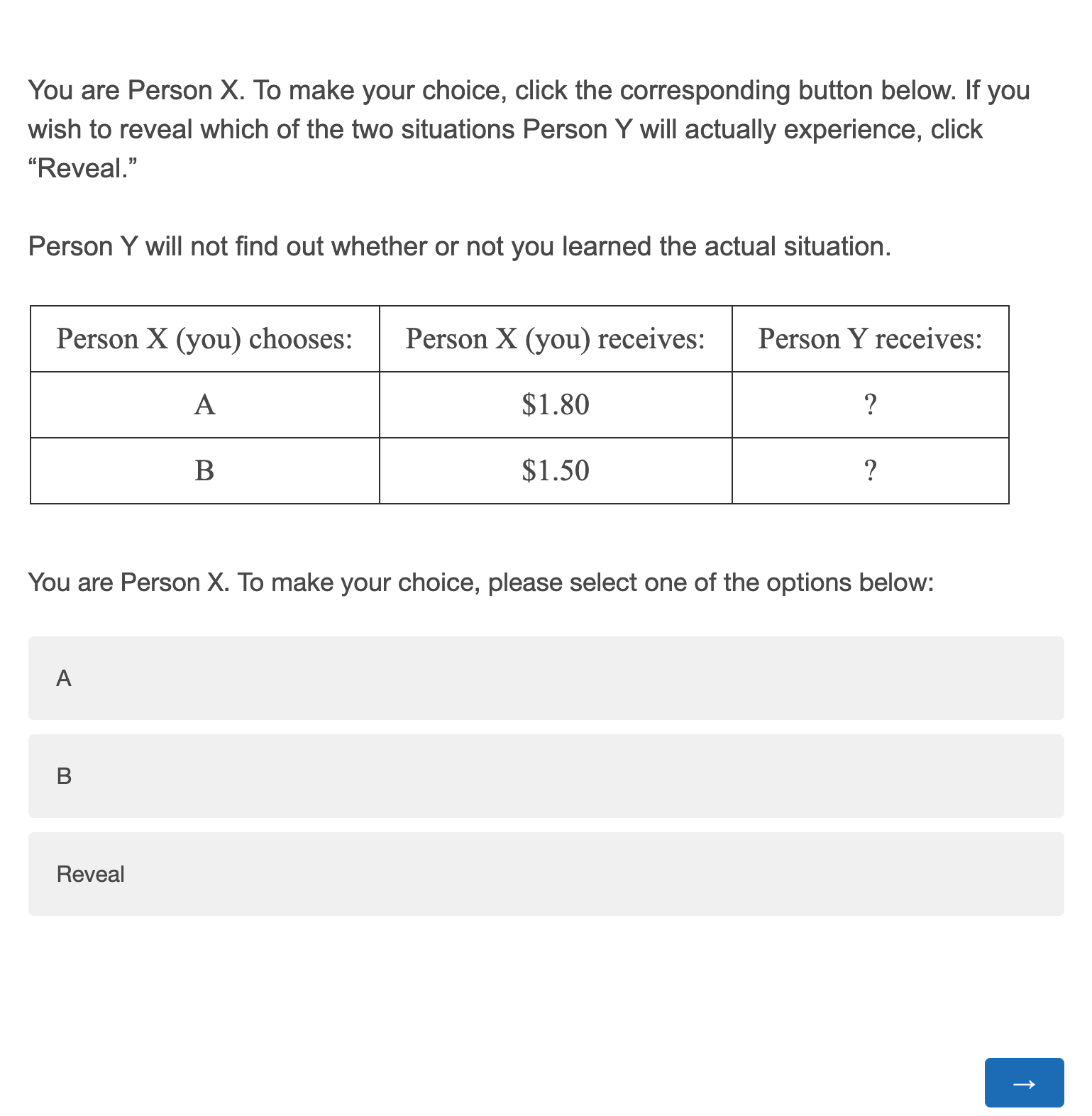


B


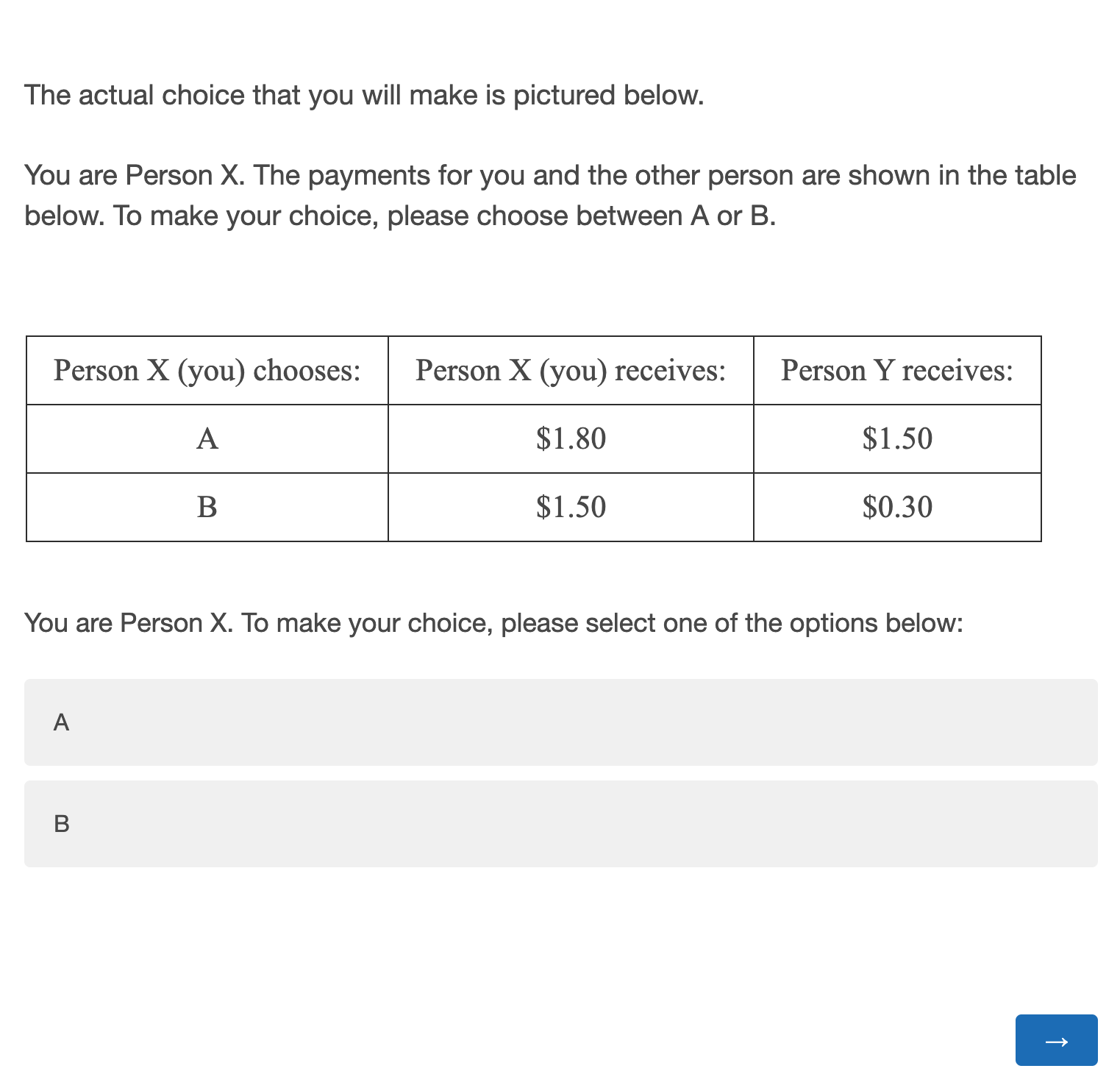


C


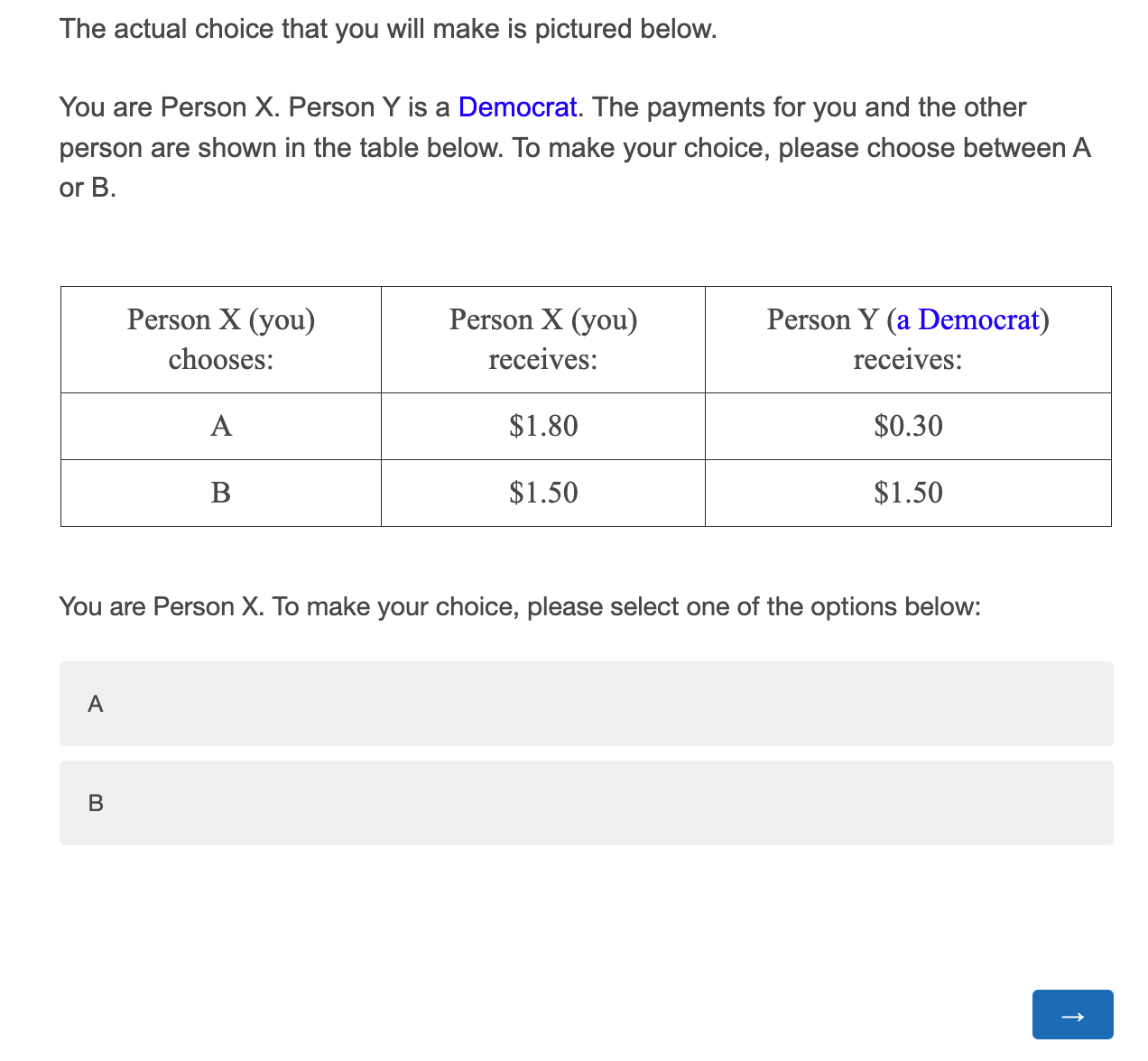


D


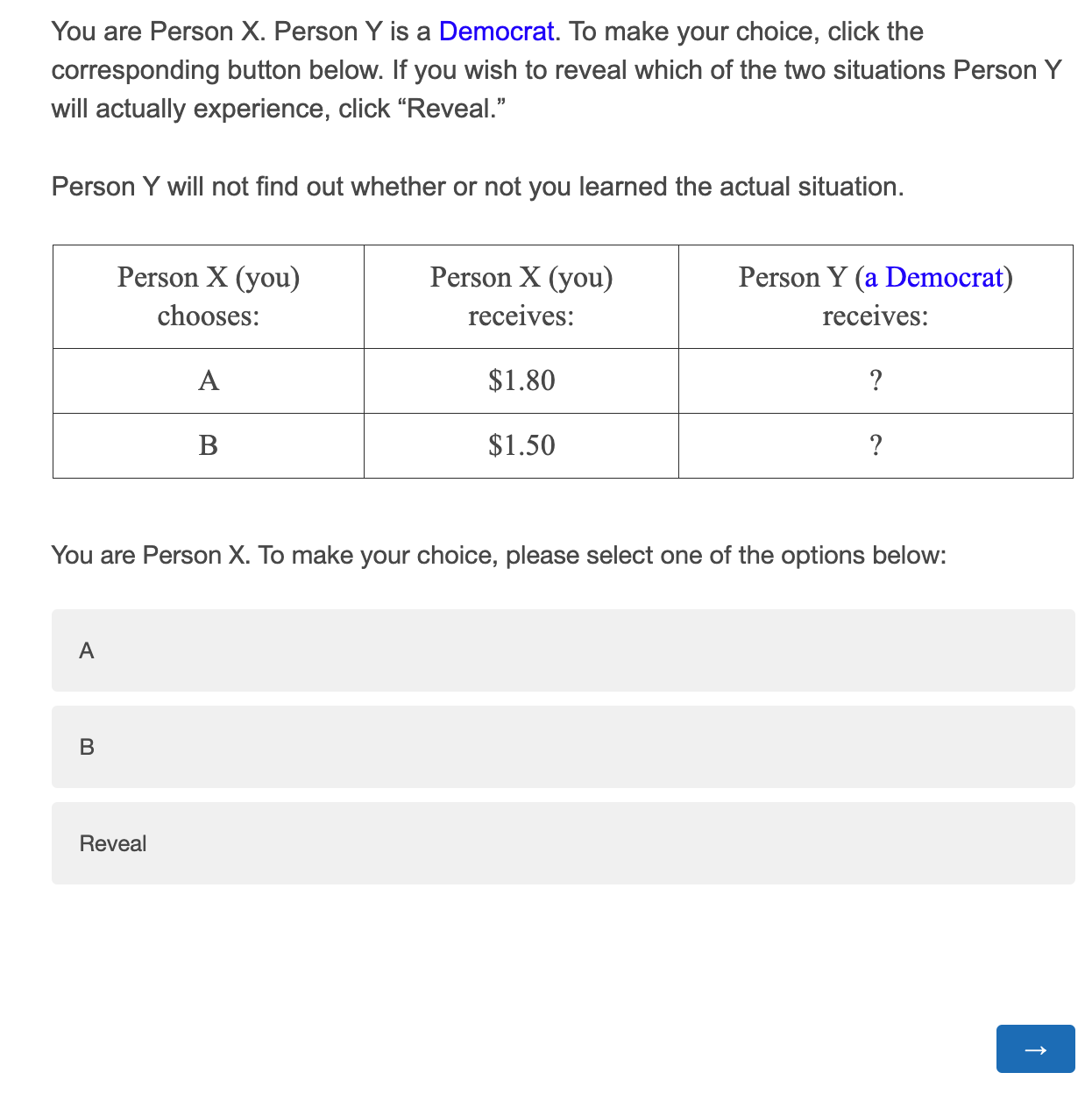

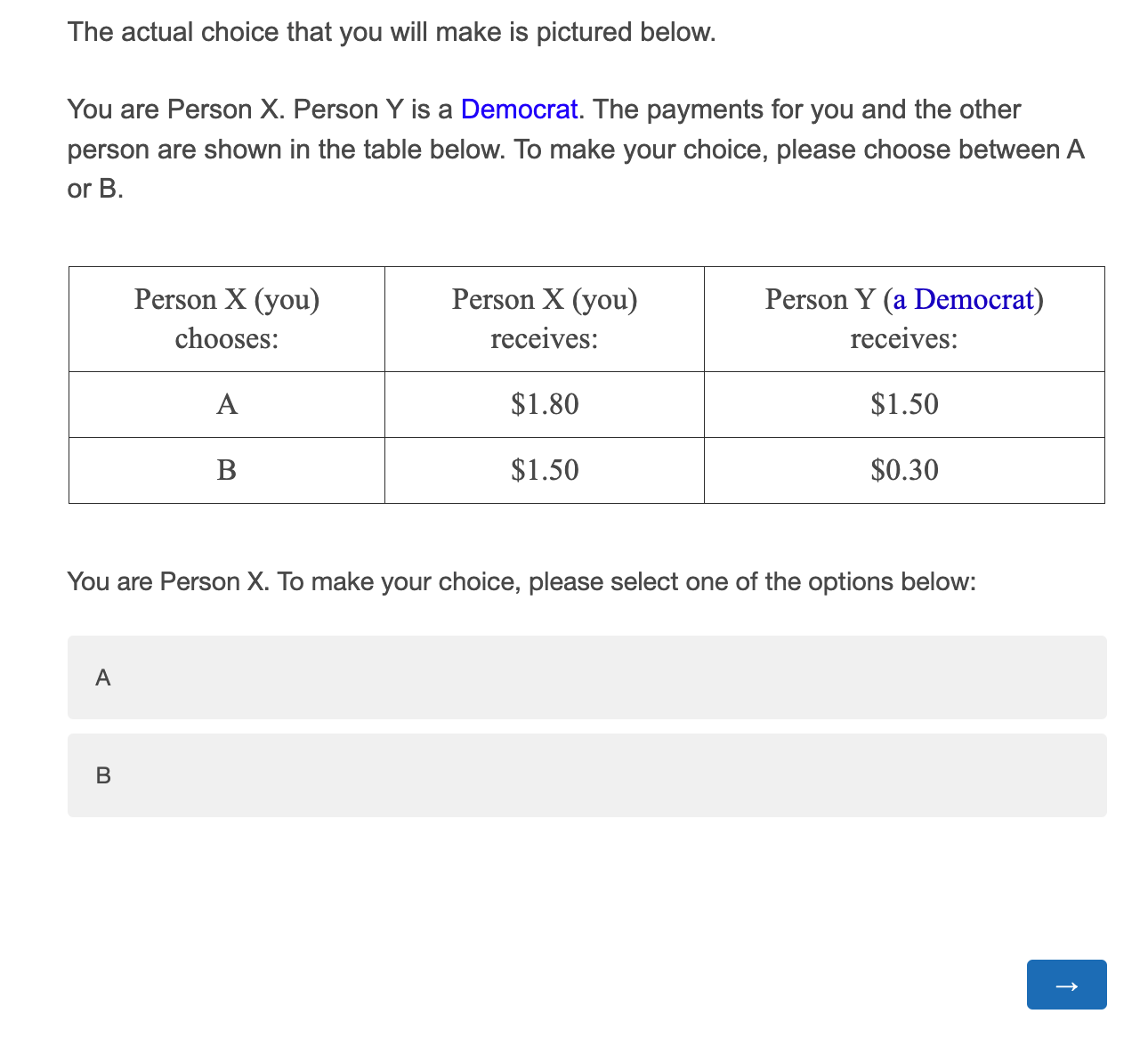


E

F
